# Association of undernutrition and female infertility in East Africa: Finding from multi-country demographic and health surveys

**DOI:** 10.3389/fgwh.2022.1049404

**Published:** 2022-12-15

**Authors:** Kedir Teji Roba, Tahir Ahmed Hassen, Tara Wilfong, Nanati Legese Alemu, Hiwot Darsene, Gelila Zewdu, Tarekegn Negese, Belaynesh Yifru, Eptisam Mohammed, Temam Beshir Raru

**Affiliations:** ^1^ School of Nursing and Midwifery, College of Health and Medical Science, Haramaya University, Harar, Ethiopia; ^2^ School of Public Health, College of Health and Medical Science, Haramaya University, Harar, Ethiopia; ^3^ School of Pharmacy, College of Health and Medical Science, Haramaya University, Harar, Ethiopia; ^4^ Food and Nutrition Case Team, Ministry of Health, Addis Ababa, Ethiopia

**Keywords:** secondary infertility, Eastern Africa, undernutrition and secondary infertility, undernutrition, DHS

## Abstract

**Introduction:**

Infertility is one of the public health problems affecting a significant number of women in the reproductive age group. Although female fertility is predominantly affected by gynecological and systemic diseases, lifestyle and nutritional factors also play an important role in secondary female infertility. Therefore, this study aimed to determine the pooled prevalence of secondary female infertility and its association with undernutrition using nationwide data from the Demographic and Health Surveys (DHS) of eastern African countries.

**Methods:**

The data of ten East African countries that comprise a weighted sample of 38,020 women data were accessed from measure DHS. Data processing and analysis were performed using STATA 15 software. A multilevel mixed-effect logistic regression model was fitted to examine the association between undernutrition and secondary infertility. Variables with a *p*-value < 0.05 were declared as significant factors associated with secondary infertility. Model comparison was done based on Akaike and Bayesian Information Criteria (AIC and BIC). To measure variation (random effects), Community-level variance with standard deviation and intra-cluster correlation coefficient (ICC) was used.

**Result:**

The proportion of women who have secondary infertility was 16.32% with 95%CI (15.96, 16.69), of which 26.94% were undernourished. This study found that being undernutrition (AOR = 1.74; 95%CI: 1.54–1.98) and overweight (AOR = 1.72; 95%CI: 1.62–1.86) were significantly associated with secondary infertility. Women aged >35 years (AOR = 3.47; 95%CI: 2.66–4.55), and rural residents (AOR = 1.16; 95%CI: 1.02–1.37) are other factors that are positively associated with secondary infertility. However, primary education (AOR = 0.87; 95%CI: 0.77–0.97) and richer wealth index (AOR = 0.84; 95%CI: 0.73–0.97) are protective factors for secondary infertility.

**Conclusion:**

This study indicated that there is a strong association between secondary infertility and undernutrition growing in Eastern Africa. Therefore, Health information dissemination and awareness creation on the impact of malnutrition on infertility should be given to the community and health care providers. Given this, it may lead to integrating nutrition counseling into both clinical settings for infertility management as well as national dietary guidelines for individuals of reproductive age.

## Introduction

Infertility is defined by the World Health Organization (WHO) as failure to achieve a pregnancy after 12 months or more of regular unprotected sexual intercourse (1). It is classified as primary infertility (when conception has never occurred) and secondary infertility (conception previously occurred at least once, but fails to repeat) (2).

Female infertility is one of the public health problems affecting a significant number of women in the reproductive age group (3). The WHO estimated that approximately 48 million couples are lives with infertility globally (4). Evidence from a recent systematic review and meta-analysis also indicated that the pooled global prevalence of female infertility stands at 46.25%, with primary infertility affecting 51.5% of women (5).

Although female fertility is predominantly affected by gynecological and systemic diseases, lifestyle and nutritional factors also play an important role in fertility (6). Malnutrition is one of the key factors associated with female infertility. Although both undernutrition and overnutrition are linked with female infertility, undernutrition is the common form of malnutrition associated with female infertility in developing nations (7) where food insecurity is highly prevalent (8). Inadequate food consumption, insufficient alimentary regimes, or general lack of nutrients are linked to female infertility by reducing body weight and physical performance, delaying puberty, lowering gonadotropin secretion level, and eventually altering the ovarian cycle (9). In addition to interfering with ovarian functions, some nutrients also affect the implantation of a normal embryo (10).

Evidence indicated that although female infertility is influenced by various nutritional factors, its epidemiology varies with geographical locations (11). For example, while it is mainly associated with eating in excess, fast food consumption, hypercaloric dietary regimens, and obesity in well-developed nations, female infertility is mainly related to inadequate dietary intake and underweight in developing nations (6). Fluctuation of the body weight in terms of overweight, obesity, or underweight, which are resulted from alterations in energy balance, is believed to produce ovulatory disorders in women. For example, it has been reported that the time to conceive is longer in women with body mass index (BMI) greater than 25 kg/m^2^ or less than 19 kg/m^2^, putting the risk of infertility on both ends of the BMI spectrum (10, 12).

Estimating the rates of infertility correctly is generally difficult, owing to factors such as imperfect measurement methods and cultural biases (13). As a result, there are limited studies indicating the regional and global pooled prevalence of female infertility (3, 5, 14). For example, to the best of our knowledge, the last time that international organizations such as the WHO estimated the global prevalence of female infertility was in 2002 (15), indicating the need for the evidence update. In addition, the association between undernutrition and female infertility is not well explored, particularly in eastern African countries where food insecurity is predominant, as studies mainly focus on gynecological and systemic factors of infertility (16, 17). This study, therefore, aimed to determine the pooled prevalence of secondary female infertility and investigate its association with undernutrition using nationwide data from the Demographic and Health Surveys (DHS) of eastern African countries.

## Methods

### Study setting and data source

According to the United Nations (UN) division, the African continent has five regions. Among these countries, East Africa includes 19 countries (Burundi, Comoros, Djibouti, Ethiopia, Eritrea, Kenya, Madagascar, Malawi, Mauritius, Mozambique, Reunion, Rwanda, Seychelles, Somalia, Somaliland, Tanzania, Uganda, Zambia, and Zimbabwe) and it is the one largest region. This study was based on Demographic and Health Surveys (DHS). Among these 19 East African countries, 13 countries have DHS data whereas 6 (Djibouti, Somalia, Somaliland, Seychelles and Mauritius, Reunion) did not have DHS data. Among these 13 countries that have DHS data, 2 countries have DHS data that was conducted before 2010 (Eritrea-2002 and Madagascar-2008) and Tanzania did not have secondary infertility cases in its recent survey. In this study, we included 10 countries' DHS data that was conducted after 2010.

The data of these 10 East African countries were accessed from the demography health survey (DHS) program official database www.measuredhs.com, after authorization was granted through an online request by explaining the goal of our study. We used the individual Record (IR file) data set and extracted the dependent and independent variables. To collect knowledge that is comparable across countries in the world, the DHS program adopts standardized methods involving uniform questionnaires, manuals, and field procedures. DHS is a nationally representative household survey that offers data from a wide variety of population, health, and nutrition tracking and effect assessment measures with face-to-face interviews of women aged 15 to 49. Stratified, multi-stage, random sampling is used in the surveys. In each country, information was obtained from qualified women aged 15 to 49 years. Detailed survey techniques and methods of sampling used to collect data have been recorded elsewhere (18). The numbers of participants in the 10 East African countries from which data were extracted are listed (Table 1).

**Table 1:** The study participants of infertility and undernutrition in East African countries.

East Africa Countries	Number of Participants
Burundi	3,759
Comoros	911
Ethiopia	7,268
Kenya	5,278
Malawi	3,822
Mozambique	2,346
Rwanda	3,074
Uganda	2,402
Zambia	5,537
Zimbabwe	3,623
**Total sample size**	**38,020**

### Population and eligibility criteria

The source population for this study was all the women aged 15–49 years in East Africa. All women aged 15–49 years and married for more than 12 months who were in the selected countries were the study population. Women who had a previous history of pregnancy and have had regular sexual intercourse without using contraception were included. However, specific women who were currently using any contraceptive method and have no measurement for the outcome interest, were excluded from this study.

### Variables

The response (outcome) variable of this study was secondary infertility. The response variable is binary and it is coded as 1 if women have been married for more than 12 months had a previous history of pregnancy, have had regular sexual intercourse without using contraception, and who have not given birth in the past 12 months and 0 otherwise.

Based on different literature, two types of independent variables were considered. Individual-level and community-level variables. Community-level variables include country and residence. The individual level variables are age, level of education, body mass index, wealth index, duration of a relationship, age at first birth, a year since last birth, Pattern of use of contraceptive, pregnancy termination, and husband's desire for children.

#### Malnutrition is derived from BMI and classified as follows

If the BMI of women is less than 18.5, we classified them as underweight/undernutrition, if the BMI of women is 18.5 to <25, we classified them as normal, If the BMI of women is 25.0 to <30, we classified them as overweight (pre-obesity), and If BMI of women is 30.0 or higher, we classified them as obesity.

### Data processing and management

Data processing and analysis were performed using STATA 15 software. The data were weighted using sampling weight, primary sampling unit, and strata before any statistical analysis to restore the representatives of the survey and to tell the STATA to take into account the sampling design when calculating standard errors to get reliable statistical estimates. Cross tabulations and summary statistics were conducted to describe the study population.

### Statistical analysis

Since the DHS data has a hierarchical nature, women within a cluster may be more similar to each other than women in the other cluster. Due to this, the assumption of independence of observations and equal variance across clusters might be violated. Therefore, an advanced statistical model is required to take into account the cluster variability to get a reliable standard error and unbiased estimate.

Furthermore, by taking into account the dichotomous nature of the outcome variable, multilevel mixed effect logistic regression was fitted. Model comparison was done based on Akaike and Bayesian Information Criteria (AIC and BIC). A mixed effect model with the lowest Information Criteria (AIC and BIC) was selected.

The individual and community level variables associated with secondary infertility were checked independently in the bi-variable multilevel mixed-effect logistic regression model and variables that were statistically significant at a *p*-value <0.25 in the bi-variable multilevel mixed-effects logistic regression analysis were considered for the final individual and community level model adjustments. In the multivariable multilevel mixed-effect analysis, variables with a *p*-value ≤ 0.05 were declared as significant determinants of secondary infertility. Intra-class correlation coefficients (ICC) were used to check whether or not the multilevel model is appropriate and how much of the overall variation in the response is explained by clustering.

Four models were fitted. Model 1 was the null model that did not include exposure variables which were used to verify community variance and provide evidence to assess random effects at the community level. Model 2 was the multivariable model adjustment for individual-level variables and model 3 was adjusted for community-level factors. In model 4, the outcome variable was equipped with potential candidate variables from both individual and community level variables.

The fixed effects (a measure of association) were used to estimate the association between secondary infertility and independent variables and expressed as an odds ratio with a 95% confidence interval. Regarding the measures of variation (random effects), Community-level variance with standard deviation and intra-cluster correlation coefficient (ICC) was used.

## Results

### Socio-demographic characteristics

A total of 38,020 women were included in the final analysis. The mean ± SD age of respondents was (31.00 ± 8.04). The majority 24,825 (65.29%) of the women lie in the age group of 20–35 years. Three-fourths of the women were rural residents 28,711 (75.52%). Nearly half of the women had a primary education 18,381 (48.35%). Most of the women included were from Ethiopia 7,268 (19.12%) and the smallest number of women included was from Comoros 911 (2.40%) (Table 2).

**Table 2:** Socio-demographic characteristics of women in East Africa (38,020).

Characteristics	Weighted frequency (%)	Secondary infertility	
		Yes (%)	No (%)
**Secondary Infertility (%)**	6,207 (16.32)	31,813 (83.68)	
**Age**			
<20	2,186 (5.75)	110 (5.03)	2,076 (94.97)
20–35	24,825 (65.29)	3,245 (13.07)	21,580 (86.93)
>35	11,009 (28.96)	2,852 (25.91)	8,157 (74.09)
**Body Mass Index (*n* = 32,061)**			
Normal	21,367 (66.65)	3,415 (15.98)	17,952 (84.02)
Undernutrition/Underweight	2,976 (9.28)	802 (26.94)	2,174 (73.06)
Overweight	5,455 (17.01)	635 (11.64)	4,820 (88.36)
Obese	2,262 (7.06)	342 (15.14)	1,920 (84.86)
**Residence**			
Urban	9,309 (24.48)	1,228 (13.19)	8,081 (86.81)
Rural	28,711 (75.52)	4,978 (17.34)	23,733 (82.66)
**Country**			
Burundi	3,759 (9.89)	1,139 (30.30)	2,620 (69.70)
Comoros	911 (2.40)	90 (9.86)	821 (90.14)
Ethiopia	7,268 (19.12)	1,427 (19.63)	5,841 (80.37)
Kenya	5,278 (13.88)	495 (9.39)	4,783 (90.61)
Malawi	3,822 (10.05)	429 (11.23)	3,393 (88.77)
Mozambique	2,346 (6.17)	610 (26.00)	1,736 (74.00)
Rwanda	3,074 (8.09)	336 (10.94)	2,738 (89.06)
Uganda	2,402 (6.32)	358 (14.92)	2,044 (85.08)
Zambia	5,537 (14.56)	938 (16.95)	4,598 (83.05)
Zimbabwe	3,623 (9.53)	383 (10.57)	3,240 (89.43)
**Education level**			
No education	6,749 (17.75)	1,612 (23.88)	5,137 (76.12)
Primary	18,381 (48.35)	3,014 (16.39)	15,367 (83.61)
Secondary	9,848 (25.90)	1,221 (12.40)	8,627 (87.60)
More than secondary	3,042 (8.00)	360 (11.84)	2,682 (88.16)
**Wealth index**			
Poorest	7,267 (19.11)	1,294 (17.81)	5,973 (82.19)
Poorer	7,838 (20.62)	1,322 (16.87)	6,516 (83.13)
Middle	7,665 (20.16)	1,277 (16.66)	6,388 (83.34)
Richer	7,779 (20.46)	1,201 (15.44)	6,578 (84.56)
Richest	7,471 (19.65)	1,112 (14.89)	6,358 (85.11)

### Reproductive characteristics

Nearly half (48.54%) of the women had their first birth at the age of 20–25 years and the majority 18,361 (48.29%) of them are currently using a contraceptive. Only 14.5% of the women terminated their pregnancies (Table 3).

**Table 3:** Reproductive characteristics of women in East Africa (*n* = 38,020).

Characteristics	Total frequency (%)	Secondary infertility	
		Yes (%)	No (%)
**Duration of relationship (years) (*n* = 33,621)**			
1	3,409 (9.60)	311 (9.11)	3,098 (90.89)
2	1,910 (5.38)	278 (14.55)	1,632 (85.45)
3	1,792 (5.05)	262 (14.65)	1,529 (85.35)
4	18,310 (5.18)	269 (14.60)	1,571 (85.40)
>=5	24,671 (69.48)	5,087 (20.62)	19,584 (79.38)
**Age at first birth (*n* = 35,773)**			
<15	12,697 (35.49)	2,115 (16.66)	10,582 (83.34)
15–19	3,626 (10.14)	735 (20.27)	2,891 (79.73)
20–25	17,366 (48.54)	2,909 (16.75)	14,457 (83.25)
25–30	1,735 (4.85)	347 (19.99)	1,388 (80.01)
>30	350 (0.98)	101 (28.89)	248 (71.11)
**Year since last birth (*n* = 27,860)**			
2	8,076 (22.58)	1,985 (24.58)	6,091 (75.42)
3	5,523 (15.44)	815 (14.76)	4,708 (85.24)
4	3,692 (10.32)	606 (16.41)	3,086 (83.59)
>=5	10,569 (29.54)	2,801 (26.50)	7,768 (73.50)
**Pattern of use of contraceptive**			
Currently using	18,361 (48.29)	0 (0.00)	18,361 (100.00)
Used since last birth	4,453 (11.71)	1,499 (33.66)	2,954 (66.34)
Used before last birth	4,086 (10.75)	1,147 (28.08)	2,939 (71.92)
Never used	11,120 (29.25)	3,561 (32.02)	7,559 (67.98)
**Last birth a caesarian-section (*n* = 27,482)**			
Yes	1,676 (6.10)	169 (10.06)	1,507 (89.94)
No	25,806 (93.90)	3,639 (14.10)	22,167 (85.90)
**Ever had terminated pregnancy**			
Yes	5,513 (14.50)	1,215 (22.03)	4,298 (77.97)
No	32,507 (85.50)	4,992 (15.36)	27,515 (84.64)
**Husband's desire for children (*n* = 37,174)**			
Both want same	17,468 (46.99)	2,633 (15.07)	14,835 (84.93)
Husband wants more	8,660 (23.29)	1,713 (19.78)	6,947 (80.22)
Husband wants fewer	3,933 (10.58)	583 (14.82)	3,350 (85.18)
Don’t know	7,113 (19.14)	1,277 (17.95)	5,837 (82.05)

### Prevalence of secondary infertility

**Figure 1:**
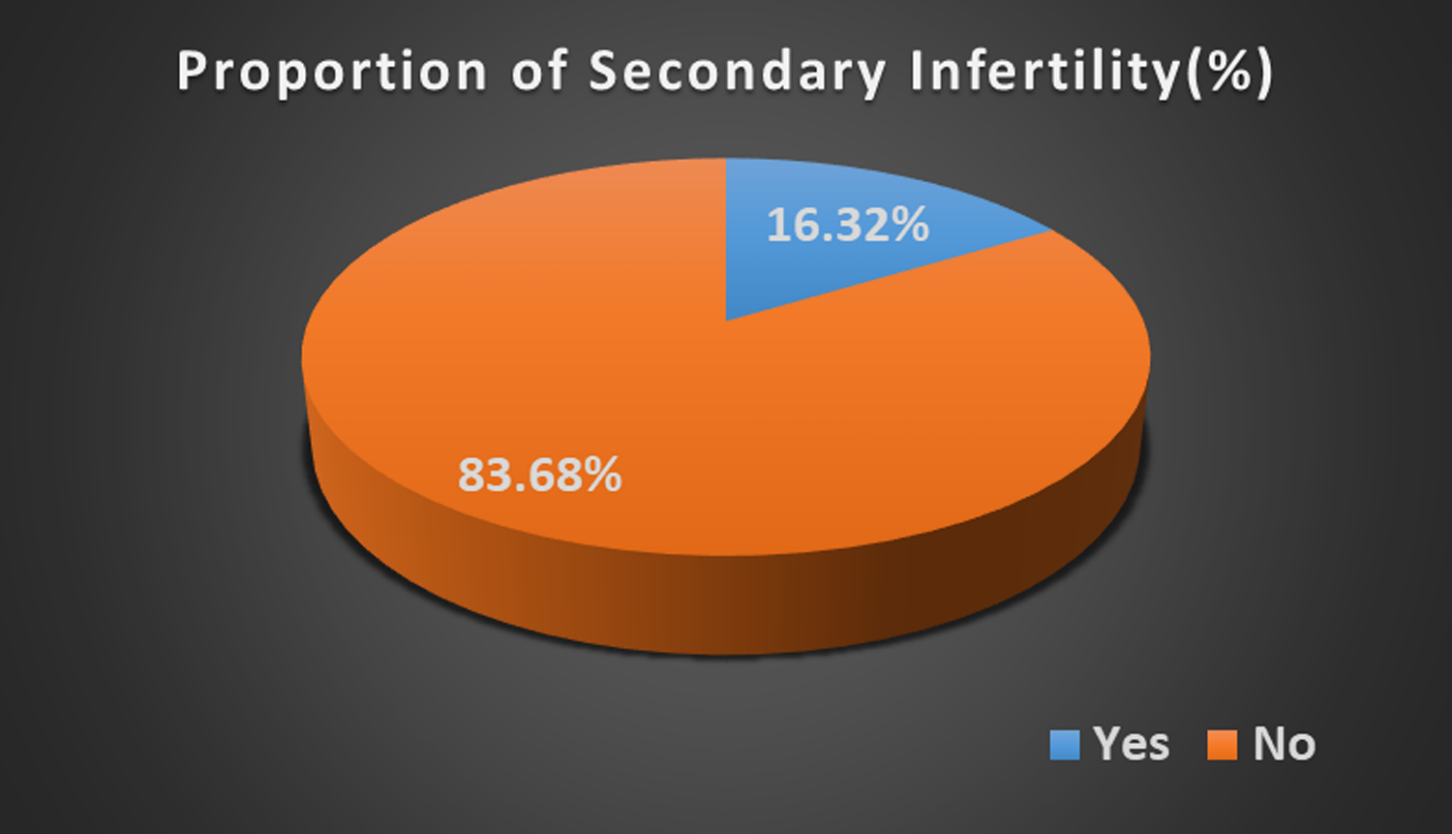
Prevalence of secondary infertility in East Africa.

The proportion of women who have secondary infertility was 16.32% with 95%CI (15.96, 16.69) (Figure 1). There is a significant association between undernutrition and secondary infertility in women. The majority 66.65% of the participants were reported to have a normal BMI, while 7.06% were obese.

### Determinants of secondary infertility

#### Result of the random effects

The null model results revealed that there was statistically significant variability in the odds of secondary infertility with a community variance of 0.76 and the ICC in the null model suggested that 18.73% of the total variability in secondary infertility was ascribed to the differences between communities. In the full model (model adjusted for both individual and community-level factors) community variance = 0.29; SE 0.02, remained significant but reduced and 6.04% of the total variance of secondary infertility can be ascribed to the community (Table 4).

**Table 4:** Multi-variable multilevel logistic regression analysis of factors associated with secondary infertility in East Africa.

Variables	Models			
	Model 1 AOR (95%CI)	Model 2 AOR (95%CI)	Model 3 AOR (95%CI)	Model 4 AOR (95%CI)
**Age of respondent**				
<20	–	1.00	–	1.00
20–35	–	1.83 (1.42, 2.34)**	–	1.89 (1.47, 2.42)**
>35	–	3.38 (2.60, 4.39)**	–	3.47 (2.66, 4.55)**
**Place of residence**				
Urban	–	–	1.00	1.00
Rural	–	–	1.20 (1.09, 1.31)**	1.16 (1.02, 1.37)**
**Highest Education Level**				
No education	–	1.00	–	1.00
Primary	–	0.72 (0.64, 0.80)**	–	0.87 (0.77, 0.97)**
Secondary	–	0.49 (0.43, 0.58)**	–	0.73 (0.62, 0.86)**
More than secondary	–	0.48 (0.38, 0.61)**	–	0.75 (0.58, 0.96)*
**Wealth index**				
Poorest	–	1.00	–	1.00
Poorer	–	0.97 (0.85, 1.00)	–	0.92 (0.81, 1.04)
Middle	–	0.94 (0.82, 1.07)	–	0.87 (0.76, 1.00)
Richer	–	0.93 (0.80, 1.07)	–	0.84 (0.73, 0.97)*
Richest	–	1.17 (0.99, 1.39)	–	0.99 (0.82, 1.20)
**Body Mass Index**				
Normal	–	1.00	–	1.00
Undernutrition/Underweight	–	1.89 (1.66, 2.14)**	–	1.74 (1.54, 1.98)**
Overweight	–	1.88 (1.50, 2.67)**	–	1.72 (1.62, 1.86)**
Obesity	–	0.66 (0.53, 1.82)	–	0.86 (0.69, 1.08)
**Age at first birth**				
20–25	–	1.00	–	1.00
≤15	–	1.21 (1.04, 1.41)*	–	1.21 (1.03, 1.41)*
15–19	–	0.98 (0.89, 1.08)	–	1.00 (0.91, 1.10)
25–30	–	0.99 (0.81, 1.22)	–	1.04 (0.85, 1.28)
>30	–	1.43 (0.95, 2.15)	–	1.53 (1.01, 2.31)*
**Last Birth (CS)**				
No	–	1.00	–	1.00
Yes	–	0.89 (0.72, 1.10)	–	0.99 (0.80, 1.22)
**Husband's desire for children**				
Both want same	–	1.00	–	1.00
Husband wants more	–	1.23 (1.10, 1.36)**	–	1.18 (1.06, 1.31)*
Husband wants fewer	–	0.93 (0.81, 1.08)	–	0.92 (0.79, 1.06)
Don't know	–	1.08 (0.96, 1.21)	–	1.05 (0.94, 1.20)
**Ever had terminated pregnancy**				
No	–	1.00	–	1.00
Yes	–	1.40 (1.24, 1.57)**	–	1.37 (1.22, 1.54)**
**Country**				
Ethiopia	–	–	1.00	1.00
Burundi	–	–	1.94 (1.68, 2.24)**	1.95 (1.62, 2.34)**
Comoros	–	–	0.45 (0.34, 0.59)**	0.44 (0.30, 0.65)**
Kenya	–	–	0.41 (0.35, 0.47)**	0.43 (0.35, 0.53)**
Malawi	–	–	0.51 (0.43, 0.59)**	0.56 ((0.45, 0.70)**
Mozambique	–	–	1.54 (1.31, 1.80)**	1.76 (1.43, 2.18)**
Rwanda	–	–	0.49 (0.41, 0.57)**	0.35 (0.29, 0.43)**
Uganda	–	–	0.71 (0.59, 0.83)**	0.90 (0.72, 1.12)
Zambia	–	–	0.86 (0.75, 1.00)	0.57 (0.41, 1.79)
Zimbabwe	–	–	0.47 (0.39, 0.56)**	0.46 (0.35, 0.59)**
**Random effects**				
Community variance	0.76 (0.04)	0.99 (0.08)	0.53 (0.04)	0.29 (0.02)
ICC%	18.73%	23.15%	13.90%%	6.04%
**Model comparison**				
AIC	32,736.01	17,274.91	31,990.62	16,844.42
BIC	32,753.01	17,458.36	32,092.64	17,099.64

ICC, intra-class correlation coefficient; AOR, adjusted odds ratio; AIC, Akaike information criteria; BIC, Bayesian information vriteria.

* Significant at *p*-value < 0.05.

** Significant at *p*-value < 0.001.

#### Result of the fixed effects

The model with smaller AIC and BIC was the best to fit the data and the interpretation of the fixed effects was based on this model. Model-3 was adjusted for both individual and community-level factors that have small AIC and BIC, compared to other models and this model fits the data well. In the multivariable analysis respondent's age group, respondent education, wealth index, body mass index, age at 1st birth, husband's desire for children, pregnancy termination, place of residence, and living country were significantly associated with secondary infertility in East Africa at a 5% level of significance.

The odds of secondary infertility were 3 times higher among the women in the age group of >35 years, compared to the women in the age group of <20 years (AOR = 3.47; 95% CI; 2.66–4.55). This suggests that the higher the age of the woman, the more likely the woman would have secondary infertility. The women who have more than secondary education levels were 25% less likely to have secondary infertility, compared to those with no formal education (AOR = 0.75; 95%CI; 0.58–0.96). Undernourished/underweight women were 74% more likely to have secondary infertility than women who are normal (AOR = 1.74; 95% CI; 1.54–1.98). Women who have pregnancy termination are 1.3 times more likely to have secondary infertility than their counterparts (AOR = 1.37; 95% CI; 1.22–1.54). Women living in Burundi and Mozambique are 1.9 and 1.7 times more likely to have secondary infertility respectively than the women living in Ethiopia; Burundi (AOR = 1.95; 95% CI; 1.62–2.34) and Mozambique (AOR = 1.76; 95% CI; 1.43, 2.18) (Table 4).

## Discussion

This study revealed that the prevalence of secondary infertility in East African Countries was 16.32%. Respondent's age group, education, wealth index, body mass index, age at 1st birth, husband's desire for children, pregnancy termination, place of residence, and living country were significantly associated with secondary infertility in East Africa.

The magnitude of secondary infertility in East African Countries was 16.32%, with the highest secondary infertility in Burundi (30.30%) and the lowest secondary infertility in Kenya (9.39%). This is in contrast to a study that analyzed the regional and global trend of infertility, in which the prevalence of secondary infertility ranged from 7.2% (5.0%, 10.2%) in the High-Income region and 7.2% (5.9%, 8.6%) in the North Africa/Middle East region to 18.0% (13.8%, 24.1%) in the Central/Eastern Europe and Central Asia region (14), a study from Iran 2.18% (1.56–2.89) (19), China 1.1%–18.04% (20), and comparable with the study conducted in United States 8.6%–27.5% (21). This might reflect the economic disadvantage of women living in the region, where infectious diseases and poor dietary practices including the consumption of antioxidant diets are the major risk factors for infertility in addition to poor access to quality health care. Therefore, awareness creation towards healthy dietary practices and weight management may be helpful in to reduce the level of infertility addition to medical therapies that induces infertility. Another causes of infertility among consumption low fat and high fiber food is also contributed to infertility in developing countries.

This study revealed that there is an association between secondary infertility and undernutrition. This result is supported by the study conducted in SSA (22) and India (23). This might be due to the reason that undernourished women had a low level of estrogen and leptin production associated with poor fat storage (9, 24). In addition, excessive workload, poor dietary intake including lack of nutrients which results in loss of both body weight and physical performance, delayed puberty, lengthening of the post-partum interval to conception, lower gonadotropin secretion levels with alterations of the physiological ovarian cyclicity and all these can have increased infertility (9, 25, 26). Therefore, dietary and weight management before preconception should be advised.

The odds of secondary infertility were higher among women of older age groups compared to women of younger age groups. This is supported by the study conducted in Kigali, Rwanda (20) and Henan Province, China (27). This might be due to the number of oocytes in the ovaries decreasing naturally and progressively through the process of atresia, resulting in a gradual but significant decrease in the fecundity of women at age 32 and a rapid decrease after age 37 (28). In addition, as the age of a woman increases, the quality of the eggs decreases as a result of an increase in the circulating level of follicle-stimulating hormone and decreases in inhibin B concentrations.

This study found a significant association between infertility and adolescent motherhood (age <15 years). This is in line with the study conducted in India (29). This might be due to the high rate of pregnancy complications at this age group (30), and unsafe abortion (31). In addition, about half of all young women are sexually active by the time they are 18 years' old and the sexual inactive nature among most of the women less than 15 years of age might contribute for higher odds of infertility (32).

The result of our study showed a negative association between the highest level of education and infertility in that as the education level of the women is higher, the odds of secondary infertility decrease. This result is contrary to a result obtained in developed countries (33). This might be due to delayed marriage, use of contraception, and better health-seeking behavior among women in developed countries (34). Conversely, educated women in developing countries are more likely to have a better income to enable them to access better healthcare services for maternity care, UTIs, or STI treatment, which are major causes of infertility in the developing world (35).

In this study, rich women were less likely to have secondary infertility than the poorest women. This is in line with the study conducted in Sub-Saharan Africa (36) and industrial populations (37). This is explained by the reason that the nutritional intake among poor women were less and this makes poor women highly infertile (38). An increase in income may be related to the consumption of healthy diets which have been associated with good fertility outcomes in women (39).

This study found that the odds of secondary infertility were higher among rural women than in urban. This is supported by the previous study conducted in India (40). This can be attributed to the fact that among the women in rural areas first child matters a lot and less importance is given to the second child; hence, they do not seek treatment also which, in turn, leads to a high prevalence of secondary infertility.

This study revealed that there is a positive significant association between secondary infertility and pregnancy termination. Women who had history of pregnancy termination were more likely to have secondary infertility. This is supported by the study conducted in Indiana (41).

Furthermore, the women living in Burundi and Mozambique are more likely to have secondary infertility than women living in Ethiopia, while the women living in Rwanda, Kenya, and Zimbabwe are less likely to have secondary infertility, compared to those living in Ethiopia. This can be due to differences in health service coverage among countries, in which countries who had higher health service coverage might have lower odds of infertility.

## Conclusion

This study revealed that undernutrition significantly increases the risk of secondary infertility for mothers, even after controlling for other socioeconomic factors. Respondent's age group, respondent's education, wealth index, age at 1st birth, husband's desire for children, pregnancy termination, place of residence, and living country were among other factors that are significantly associated with secondary infertility in East Africa. The strong association of women's education and household wealth with secondary infertility highlights the need for efforts to improve households' livelihoods and increase girls' schooling to correct perceptions of the importance of skilled maternal health care. Therefore, health information dissemination and awareness creation on the impact of undernutrition on infertility should be given to the community and health care providers.

## Data Availability

Publicly available datasets were analyzed in this study. This data can be found here: The data we used for this analysis is publicly available through the MEASURE DHS program, and you can access it from www.measuredhs.com after explaining the objectives of the study. The data is then accessible can be freely downloaded after receiving the authorization letter.
